# Preferences for an Experience Sampling Method–Based Tool as an Adjunct to Usual Treatment in Patients With Problem Substance Use: Qualitative Study

**DOI:** 10.2196/79510

**Published:** 2026-01-15

**Authors:** Adam Kurilla, Natália Čavojská, Theresa Ikegwuonu, Marta Nemčíková, Julia CC Schulte-Strathaus, Lotte Uyttebroek, Joanne R Beames, Dagmar Breznoščáková, Daniel Dančík, Michal Hajdúk, Anton Heretik, Ľubomíra Izáková, Zuzana Katreniaková, Inez Myin-Germeys, Ján Pečeňák, Ulrich Reininghaus, Anita Schick, Maria Wolters

**Affiliations:** 1 Department of Psychology Faculty of Arts Comenius University Bratislava Bratislava Slovakia; 2 Centre for Medical Informatics Usher Institute University of Edinburgh Edinburgh United Kingdom; 3 Department of Social and Behavioural Medicine Faculty of Medicine University of Pavol Jozef Šafárik Košice Slovakia; 4 Department of Public Mental Health Central Institute of Mental Health Heidelberg University Mannheim Germany; 5 Center for Contextual Psychiatry Department of Neurosciences KU Leuven Leuven Belgium; 6 Department of Psychiatry Faculty of Medicine Comenius University Bratislava Bratislava Slovakia; 7 Health Service and Population Research Department Institute of Psychiatry, Psychology & Neuroscience King's College London London United Kingdom; 8 Partner site Mannheim‑Heidelberg‑Ulm German Center for Mental Health (DZPG) Mannheim Germany; 9 ESRC Centre for Society and Mental Health King's College London London United Kingdom; 10 School of Informatics University of Edinburgh Edinburgh United Kingdom

**Keywords:** mHealth, Experience Sampling Method, Ecological Momentary Assessment, digital mental health, user preferences, substance use, qualitative research

## Abstract

**Background:**

Mobile health tools that use the Experience Sampling Method (ESM) appear to be a promising tool to streamline and improve the treatment of substance use disorders. However, patient involvement in the development of these tools is uncommon, and research on the preferences of people being treated for substance use disorders has been scarce. In the scope of the European Union IMMERSE (Implementing Mobile Mental health Recording Strategy for Europe) consortium, an ESM-based tool for Digital Mobile Mental Health (DMMH) was first codeveloped and later tested in 4 European countries.

**Objective:**

This study aimed to achieve an understanding of preferences for features of DMMH among mental health service users with problem substance use.

**Methods:**

In 4 European countries, service users were recruited for a semistructured qualitative interview, which started with the presentation of a prototype of the DMMH. Data analysis was performed through directed qualitative content analysis.

**Results:**

The analytical sample consisted of 12 (5 female, 6 male, and 1 nonbinary person) participants with problem substance use aged 18-50 years. There were 4 participants from Slovakia, 3 from Belgium, 4 from Germany, and 1 from Scotland. Patient preferences were classified into 7 categories: notifications, questions, user interface, functionality changes, visualizations, sharing data with clinicians, and sharing data with others. The proposed intensive notification schedule was deemed acceptable by service users as long as the questionnaire is short. Participants expressed a preference for open-text response options, Ecological Momentary Interventions, and options for individual customization of several elements of the tool. Data visualization was considered an important aid for communication with clinicians, with whom all participants wanted to share data obtained with DMMH. The possibility of sharing data with other people depended on the quality of the relationship with them.

**Conclusions:**

In the development of ESM-based mobile health tools for people with problem substance use, their preferences for content, functionality, and appearance of the tools should be considered so they match patients’ treatment needs.

## Introduction

Mobile health (mHealth) tools based on Experience Sampling Method (ESM) appear to be a promising element in delivering more person-centered mental health care [1-3]. mHealth is a public health practice supported by mobile devices such as mobile phones, tablets, portable patient monitoring devices, personal digital assistants, and other wireless devices [4]. ESM is a data acquisition process characterized by repeatedly sampling participants’ actual behaviors and experiences in real time in their natural environment [5]. The goal of ESM is to minimize the memory biases that arise from retrospective assessment of experience and mental states, to maximize ecological validity, and to enable capturing microprocesses that influence behavior in real-world contexts [6]. The use of ESM as part of a treatment appears to be a promising avenue toward personalized, person-centered mental health care [7]. It can help patients better understand their adaptive and maladaptive behavior patterns and the reasons behind them. Providing patients with these tools allows them to become more active partners in the treatment process [8].

In the treatment of substance use disorders (SUDs), in addition to the aforementioned benefits, ESM can make a significant contribution to the early detection of impending relapse [9] and provide a reliable basis for individually tailored interventions based on the dynamic changes during the treatment [10]. Treatments provided in mental health settings are often ineffective for patients with SUDs [11], which may be related to the fact that treatment of SUDs and comorbid disorders requires high intensity and emphasis on psychosocial interventions [12]. Relapse is preceded by craving, the intensity of which fluctuates dynamically and is often triggered by external and internal factors. ESM is a good tool to catch it early [9,13,14]. Although we have limited evidence on the efficacy of mHealth tools in the treatment of SUDs [15], some methodologically rigorous studies have produced results on their effectiveness and usefulness in increasing the number of abstinence days both during treatment and in follow-up [16-18]. A challenge to the effective application of mHealth tools in the treatment of individuals with SUDs is to explore how to successfully implement them into clinical practice. Successful and sustainable implementation of new digital health technologies is challenging and depends on a variety of factors, including patient acceptance and expectations regarding the adoption and use of these tools [19]. The feasibility and acceptability of using digital instruments with embedded ESM in the treatment of SUDs appear to be high [20-22], and this is also true for tools with intensive data collection schedules [23,24]. However, low uptake and limited user engagement are common challenges observed in digital interventions within real-world care settings [25].

A systematic review conducted by Szinay et al [26] identified 3 categories of factors that influence the uptake and effective engagement in the use of mHealth tools: capability-enhancing factors, opportunity-enhancing factors, and motivation-enhancing factors. The first category relates to app literacy skills, app awareness, available user guidance, health information, statistical information on progress, well-designed reminders, features to reduce cognitive load, and self-monitoring features. The second category includes factors such as availability at low cost, positive tone, personalization factors, recommendations for health and well-being, embedded health professional support, and social networking opportunities. Finally, the third category entails factors such as positive feedback, available rewards, goal setting, and the perceived utility of the tool. Another important factor influencing the uptake and effective engagement with digital tools is their appropriateness for the intended users and the context in which they are to be implemented. Tools are more likely to be adopted when they are personally relevant and can be seamlessly integrated into users’ daily routines [27]. A common reason for poor fit is the insufficient involvement of patients, stakeholders, and other end users in the development process. Stakeholders are typically engaged only in the final stages of development, such as during usability testing, when the opportunity to make substantive changes is limited [28,29]. Early and continuous involvement of stakeholders is essential to ensure that digital tools are aligned with real-world needs, thereby facilitating more effective implementation [30]. Qualitative research methods are central to understanding how to improve user uptake and engagement with mHealth tools [31].

Previous research on potential users of mHealth tools with SUDs has shown that positivity, personalization, monitoring of use, gaining insight into use, daily tips, and interactive features are considered facilitators of the use of mHealth tools. On the other hand, repetitiveness, receipt of nontailored content, and inability to self-navigate to desired content are considered barriers to the use of mHealth tools by individuals with SUDs [21,32,33]. Daily notifications can substantially impact the engagement of individuals in both positive and negative ways. Optimizing the notification policy to target both effectiveness and engagement is needed [34]. Although the general facilitators and barriers of mHealth tools for persons with SUDs are known, it is unclear whether and how these patterns manifest in a population with more complex needs, such as people with comorbid mental disorders.

Therefore, this study aimed to achieve a better and more detailed understanding of the preferences of mental health service users with problem substance use regarding the features of an ESM-based digital tool. More specifically, we aimed to explore what patients with problem substance use consider to be a satisfactory design (content, appearance, and functionality) of an ESM-based mHealth tool that they would use as part of their treatment.

## Methods

### Study Design and Research Paradigm

This study used a qualitative design using semistructured interviews, guided by an interpretivist-pragmatic paradigm [35], combining the interpretive understanding of participants’ experiences with a pragmatic focus on the applicability of findings to clinical practice. Semistructured interviews were designed to collect information about relevant contextual factors within the Non-Adoption, Abandonment, Scale-Up, Spread, and Sustainability (NASSS) framework [19] related to the potential use of the Digital Mobile Mental Health tool (DMMH) as a part of the IMMERSE (Implementing Mobile Mental health Recording Strategy for Europe) project [36].

### Setting and Context

The study was carried out in the context of the ongoing IMMERSE project. The IMMERSE project is dedicated to advancing person-centered mental health care through the development and implementation of the DMMH [2,36].

The DMMH consists of two components: (1) MoMent app (movisens GmbH) for patients, an ESM-based digital mobile app for systematic monitoring of patients’ current mental state, mood, symptoms, activities, context, therapy goals, key problem areas, and momentary quality of life; and (2) a dashboard that allows clinicians to (a) customize individual treatment goals and questionnaires presented by the MoMent app and (b) produce automated reports that provide meaningful information from self-assessment data using the integrated dashboard, which is an interface to visualize and elaborate the collected data into customized feedback for patients and their clinician.

This paper reports on findings from Phase I of IMMERSE, where qualitative, semistructured interviews were conducted prior to the development of the DMMH tool and its implementation (Phase II). The aim of Phase I of the project was to develop, optimize, and examine in detail the contextual factors, processes, and strategies for the implementation of the DMMH intervention in routine mental health care in 4 European countries (Belgium, Germany, Scotland, and Slovakia). It involved conducting semistructured interviews with 4 groups of stakeholders, namely service users, clinicians, people from the service user support network, and health care administrators.

In this study, we focus on service users with problem substance use. In other groups of stakeholders, relatives of service users with problem substance use, clinicians specializing in this group of patients, and health care administrators for people with SUDs were underrepresented, so we do not focus on these groups of stakeholders.

Participants for the study were recruited from multiple clinical sites across participating countries. The target sample sizes for the interview component were 40 service users (10 per country). These were users of psychiatric services in outpatient and inpatient care. Clinical sites were located in Belgium (KU Leuven University Hospital and Psychiatric Center Sint-Kamillus, Bierbeek), Germany (Central Institute of Mental Health Mannheim and Psychiatric Centre Nordbaden Wiesloch), Slovakia (Psychiatric Clinic at University Hospital Bratislava plus outpatient clinics in Vranov nad Topľou/Hanušovce/Košice), and Scotland (NHS Lothian, including Lothian CAMHS).

### Participants and Sampling

Participants were recruited using purposive sampling to ensure diversity of perspectives across treatment settings. Inclusion was based on predefined eligibility criteria relevant to the study’s aims.

Inclusion criteria for service users were (1) aged 18-64 years, (2) seeking help for mental health issues and needing specialist assessment, (3) in contact with or awaiting local mental health services, and (4) able to give informed consent. Exclusion criteria were (1) psychiatric symptoms due to an organic cause (ICD-10 [*International Classification of Diseases, Tenth Revision*] F00-F09), (2) intellectual disability or severe psychiatric disorder (*ICD-10* F71-F89) impairing consent ability, (3) medical or psychological contraindications, inability to use a smartphone, insufficient language, or terminal illness. Given the aims of this study, we focused on service users who were problem users of psychoactive substances, that is, those who had a SUD or whose substance use caused them significant problems in their lives. Participants meeting the narrower criteria for inclusion in this paper were retrieved from the interview transcripts using the following search terms: “drugs,” “alcohol,” and “addiction.” The identified transcripts were reviewed to determine whether the participants met the stated criteria.

### Recruitment Procedure

Recruitment was conducted through a combination of strategies tailored to each site. At the Belgian sites, recruitment lasted approximately 4 months (late January 2022-late May 2022), using flyers, posters, email invitations, and contacting previous participants from related studies and patient organizations. At the German sites, recruitment spanned 6 months (late January 2022-late June 2022) and used clinical site contact, patient organizations, email invites, and on-site presentations. For the Slovak sites, recruitment lasted from December 2021 to May 2022. Initial clinical-site recruitment was supplemented by private clinics, psychiatric practices, civic associations, and personal contact with patients and their supporters. The Scottish site used posters, flyers, and email invitations beginning January 2022, and later made use of third-party registers (eg, Scottish Health Research Register [SHARE] and Scottish Mental Health Research Network [SMHRN]) when initial recruitment channels proved insufficient. Recruitment ceased when minimum targets were achieved or no further participants were required.

### Data Collection

Semistructured interviews were conducted by several trained researchers across participating clinical sites. All interviewers had previous training in psychology. Not all interviewers were coauthors of this manuscript; however, all received standardized training and followed a common interview protocol to ensure methodological consistency. The interviewers were supervised by (MW and TI), who also monitored adherence to the study guide and provided regular feedback during data collection.

Participants were interviewed either in person at the clinical site or via video call, depending on their preference. Interviews were recorded using an encrypted audio recorder with the written and verbal consent of the participants. The interviews were transcribed and coded in the local language at the sites where they were conducted.

Service users’ views on notifications, questions, user interface, functionality changes, data visualizations, sharing data with clinicians, and sharing data with others and any other topics related to the design of the DMMH prototype and its use that emerged from the interviews were analyzed. We developed interview scripts that were used for data collection across all 4 participating countries (Multimedia Appendix 1). After informed consent, participants were informed about the purpose and how the DMMH is supposed to work; participants were shown prototypes of the MoMent app items and different visualizations of longitudinal patient data (Multimedia Appendices 2 and 3). Then participants were asked to share their feedback on the prototypes and how these could be implemented in their treatment. The interview guide comprised 23 questions, focusing on participants’ experiences with digital health tools, feedback on the DMMH prototype, perspectives on DMMH usability and its potential impact on the treatment, and potential barriers and facilitators of the use of such a tool. Additionally, vignettes with service user case examples were presented and discussed.

### Data Processing

Preliminary processing of interview data to derive structured data items was performed using software designed for qualitative data analysis, namely MAXQDA (VERBI Software GmbH) and NVivo (Lumivero LLC).

Quotes used in this study were translated from the original language into English using DeepL software (DeepL SE) and checked by a native speaker for accuracy.

### Data Analysis

The analysis followed a directed (top-down) qualitative content analysis approach [37,38], which starts with codes that are derived from a predefined theory (NASSS framework) [19]. The initial coding using anchor samples was done by local researchers speaking the native languages and throughout multiple sessions across countries, comparing understanding of individual codes, assurance has been achieved that all codes were interpreted in the same way. During this process, some codes were merged, omitted, or added, and the final codebook was created. This was followed by the main analysis. This first level of coding (using the NASSS framework) conducted by 2 coders per country represented the primary analysis reported elsewhere [39], while we carried out a secondary, bottom-up analysis conducted by AK and NČ focusing on notifications, questions, user interface, functionality changes, data visualizations, sharing data with clinicians, sharing data with others, and any other topics related to the design of the tool and its use in general, and ESM data in particular. This was done by enriching the existing codes with new, more detailed subcodes that emerged from the analysis.

### Techniques to Enhance Trustworthiness

To enhance the trustworthiness of the analysis, peer debriefing was carried out between 2 researchers (AK and NČ) to ensure consistency and credibility of interpretation. Emerging codes and themes were reviewed and discussed to challenge assumptions, refine interpretations, and ensure that findings accurately reflected participants’ perspectives. Consensus among team members was reached before finalizing the structure of categories.

### Researcher Reflexivity

Some of the interviews were conducted by researchers who had previous clinical contact with the participants in the context of their routine treatment. This familiarity facilitated rapport and openness during the interviews but was also acknowledged as a potential source of bias. To minimize its influence, interviewers followed a standardized interview guide and adhered to reflexive practices throughout data collection.

### Ethical Considerations

Applications to each country’s Ethics Review Board were coordinated using a joint protocol. Ethical approval was granted for all sites, in Belgium by Ethics Committee KU Leuven (S65990; January 11, 2022), in Germany by Ethics Committee II of Heidelberg University at the Mannheim Medical Faculty (633-21; September 24, 2021), in Scotland by South Central—Hampshire A Research Ethics Committee (REC reference: 21/SC/0397, IRAS project ID: 307153; December 23, 2021), and in Slovakia by Ethics Committee of the Faculty of Medicine, Comenius University and University Hospital Bratislava, Staré Mesto Hospital (115/2021; October 25, 2021) and Ethics Committee of the Faculty of Medicine, Pavol Jozef Safarik University in Kosice (1N/2022; January 27, 2022).

Participants received detailed written and oral information about the project as well as a comprehensive informed consent document.

The data collected during the interviews were pseudonymized and stored locally at the locations where the interviews took place. All subsequent data processing, including transfer to the IMMERSE project research database, was carried out using this pseudonymous identifier. The data are stored on a General Data Protection Regulation (GDPR)–compliant server at the University of Heidelberg’s computing center. The data elements derived after qualitative analysis do not contain any directly identifying data.

Participants were compensated for their time spent participating in the interview with €10 (US $11.60) in European Union countries and £10 (US $13.40) in Scotland.

## Results

### Participants

Of the 51 interviews, 12 participants met the narrower inclusion criteria for this study. The basic characteristics of the study sample are in Table 1.

**Table 1:** Overview of participants (N=12) included in a qualitative study exploring preferences for an Experience Sampling Method–based tool as an adjunct to usual treatment among patients with problem substance use.

Participant ID	Sex	Age group (years)	Country	Employment status	Education	Diagnosis
P1	Male	31-40	Slovakia	Employed	Secondary education	Substance use disorder
P2	Male	41-50	Slovakia	Employed	Secondary education	Substance use disorder
P3	Male	31-40	Slovakia	Employed	Graduate degree	Substance use disorder
P4	Male	31-40	Slovakia	Not working	Vocational qualification	Substance use disorder
P5	Female	41-50	Scotland	Not working	Primary	Schizophrenia
P6	Nonbinary	41-50	Belgium	Employed	Graduate degree	Substance use disorder
P7	Female	41-50	Belgium	Not working	Primary	Depression
P8	Female	41-50	Belgium	Employed	Graduate degree	Substance use disorder and borderline personality disorder
P9	Male	18-30	Germany	Not working	Vocational qualification	Schizophrenia
P10	Female	18-30	Germany	Employed	Secondary education	Substance use disorder, ADHD^a^, and borderline personality disorder
P11	Female	18-30	Germany	Employed	Secondary education	Substance use disorder, depression
P12	Male	31-40	Germany	Not working	Primary	Substance use disorder, ADHD

^a^ADHD: attention-deficit hyperactivity disorder.

### Main Findings

Seven categories were used from the original codebook and 8 subcategories were identified (Table 2).

**Table 2:** Main categories and subcategories were identified through directed content analysis of semistructured interviews.

Categories	Subcategories
Notifications	Number of notifications per day Questionnaire availability time
Questions	Adding questions or answer options Questionnaire length
User interface	—^a^
Functionality changes	Interventions Reminders Gamification Customization
Visualizations	—
Sharing data with clinicians	—
Sharing data with others	—

^a^Not applicable.

#### Notifications: Number of Reminders Per Day

Participants who were employed considered 8-10 notifications per day to be excessive and suggested 3-5 notifications per day.

That does make sense, but it's also a lot. I mean, if I think about it, my typical day runs from around 7:30 a.m. to 10 p.m. Getting 10 notifications in that time is just a lot. ... I think realistically, something like 3 to 5 would make more sense.

P10

Those unemployed or hospitalized at the time considered 8-10 notifications per day feasible, even something that could help them structure the day. Also, even those who found 10 notifications too many at first would find this number acceptable if the questionnaire took a short time to complete.

I actually think it's good, so for me it's not too much. For others, it might be too much because- well, I'm currently unemployed, I have time, I can answer the questions. But there are people who work, and they can't really do that.

P9

Between 7 and 10 times a day? Ah yes, that seems like a lot, yes. And when then for example? Is that then, I'm just saying, every hour or what does that mean? Every two hours? ... [Interviewer: So that's actually, we usually count 2 minutes for a questionnaire.] Ah oh yes, then that's not a problem.

P8

It was also suggested to adapt the number of notifications to the patient’s condition and stage of treatment.

The app would have to be taught somehow, in some way, to be adaptable to a given rhythm, or I don't know, because the patient would then have to essentially subordinate to the app in their life. Maybe two levels, a level for someone who has to adapt because the information is needed at these intervals, and it occurs to me that someone who has less of a problem, so that's where it could be adapted to them [number of notifications].

P2

#### Questionnaire Availability Time

Participants indicated that postponing a questionnaire, so that it could be filled out at a later time point, would be desirable and facilitate completion. The time window is preferred to be from 15 minutes to 1 hour.

Well, postponing it would be acceptable, I would like that. For example, I cannot do it now because I have a lot of work to do, and I postpone it for an hour that I know I have to complete something, and within an hour one finds those 5 minutes to complete it.

P4

#### Questions

Participants appreciated the brevity and clarity of the questions used in the DMMH. They commented that it would be helpful if the situation they were in could be described more specifically, to capture the thoughts they had in the situation, and possibly what they did to cope with it.

The questions are actually clear in what they're about, and that's already a good thing.

P12

Yeah, if I'm tense because it can catch me in the moment, if I'm arguing with somebody, so if I'm feeling tense or how I'm actually reacting when I'm tense or something more specific. Something else I would add in there would be questions like how I handled that tension or how did I handle that situation where I felt bad and why did I feel good...something came up, something along those lines. Such lighter questions, whether I'm succeeding or failing to meet the daily plan and such questions. ... Watch my thoughts, but I don't know how to do that, because those feelings are probably one of the most important, but those thoughts are important too, I find it important to watch my thoughts too. So was I thinking of drugs now, or was I thinking of alcohol, an accomplice from the past. For us addicts, I think it's a recurring thing, that it's about the same thoughts, that I've walked down the street where I used to do drugs and it automatically comes back to me, so that's where you go back to that past - how many times a day I remembered it, or something like that.

P4

#### Adding Questions or Answer Options

Participants were informed that there were planned questions about substance use and craving. They suggested adding questions about different additional issues that were relevant to them, such as eating frequency and physical activity. Suggestions for better specification of context and activities were repeated.

But the eating, for example, I have a problem with that now. With gaining weight, and I would very much like to have a regular diet. To have maybe even a reminder, like my therapist said, like for 10 or brunch, because I'm totally skipping that and I'm doing it wrong, so that would be a great thing to have a reminder, but also to follow up on. For example, some sort of note to register that I've eaten lunch at that time, I'd welcome that.

P2

Maybe those options [answers about context], it seemed to me, what I only saw cursorily, that maybe there weren't enough of them, that maybe even more detail could have been broken down, that there was only the exterior, maybe even more precise, that where I was could have been there, at least a theme or something, but that's just kind of my idea. Yeah, it seems to me exactly that I would specify it more as to whether it's some kind of nature or I'm thinking...the city centre, the built-up area where there are more people or a nature, that what is the element that's affecting that person. Also outdoors, I also seek out different environments depending on my mood and what I need.

P2

#### Questionnaire Length

Some participants were willing to dedicate 5-10 minutes to one questionnaire but preferred shorter durations, for example, 2-3 minutes.

So, 5 to 10 minutes. Anything in between is perfectly okay. [Interviewer: Okay. Yeah wow, so the idea was that it's even just 3 to 5.] Which is probably even better for the majority of people who would use that, because if it takes too much time, then you're more willing not to do it and just answer “larifari” [sloppy], sort of, as long as it's quick.

P11

Two to three minutes, certainly no longer. But two minutes that goes at work. Ten minutes you shouldn't come off with.

P6

#### User Interface

There were repeated requests for a more colorful environment and clearer color differentiation of items from the background. Some participants preferred to see multiple questions, ideally all at once, rather than one at a time. Using an avatar was an acceptable option.

In theory, maybe you could make the background a bit more colorful, or at least the bubbles - just to make them stand out a bit more. You know, it's a bit monotone, I'd say.

P12

Yeah, the avatar's nice, I think it's nicer because it's taken in a playful way a little bit, so it's closer to people I think, it's closer to me personally, the avatar.

P1

#### Functionality Changes

In addition to a randomized schedule of questionnaires, participants also suggested a questionnaire that they could recall on demand, supplemented by the possibility of recording a note in the form of text. Two participants also suggested fixed morning, lunchtime, and evening questionnaires to fit in with their day’s schedule.

Or, if I have some kind of little ache or something, then I can click somewhere in the app on “Menu” and then kind of save “Notes” somewhere on the side or something like that. … You do have those note things normally on your phone, but you usually don’t use them, but, if you're already in the app, then maybe yeah. … Or you enter it in the calendar, and then you write a side note: “Had something happen today.” Or I don’t know. “Today this and that happened.” … you click on “Menu” and then you can still write a note, kind of in the background where the scale was - where it was bad, let’s say - if you had a bad day, then you could write something right there. Because, otherwise, you only kind of know what happened, and like this, you write on that day “Ah that really annoyed me” or “I managed it”, or whatever. Or something especially positive too. … you can just tell yourself: “Hey, I’m not doing so well right now, so I’ll just write something down.” Because, for example, if the rhythm is every two hours, but now it’s already after an hour or half an hour after something happened - something went wrong, and you’re just really like [feeling] “baaah” - then you’re not gonna wait one and a half hours until some kind of “bing” [notification prompt] comes again.

P12

You have a lot of other duties, I wouldn't mind for example during shopping, but I do manual work, I'm used to doing manual work and if I'm on the line and I run out two times on the app to fill it, the manager probably wouldn't praise me that what I'm doing all the time with that phone. Before work or before breakfast if I filled it out that how did I sleep let's say or how was my morning, at lunch it would ask me how my morning was, how am I feeling at the moment, in the afternoon or in the evening to do an inventory of the day, 3-4 times I would rate it that's just right.

P4

#### Interventions

The most commonly suggested addition to the app was the delivery of simple interventions informed by ESM data. Motivational quotes, grounding techniques, coping recommendations, crisis plans, or helpline contact were suggested.

Like there were just questions though, you might just be asking about that, but that maybe there would have been some solution to that as well. Or maybe something I would have suggested that something works for me, like I don't know... I'll give you an example, take five breaths, or be present, or I don't know.

P3

And maybe somewhere too, and this may be far fetched, but if things are really not going so well and they ask in the evening “What have you been up to?”, this and that, and you're still like “Yes, I'm really not feeling well” and that's certainly going to be the case with some people, yes, that you still have a number somewhere or something you can call. Even if it's just including the Suicide Hotline. That's maybe not ideal, but it can be somewhere [in the app]. Suppose it doesn't go well, what can I do? “You don't feel well. Here are some people you can reach” and then the basic instances and their number.

P8

#### Reminders

Participants also indicated that they would use the app as a tool to remind them of other issues related to treatment and healthy lifestyle, such as taking medication, proper nutrition, and fluid intake.

Yes, I think sleep and maybe food and drink as well, because yes, I have moments when I have very little structure, and I know that's kind of common here often with people that they don't eat 3 times a day.

P8

#### Customization

There was a request for the ability to customize the color and number of notifications.

It could be that way, everyone could customize it with their own colour, we all like different colours, we all like different things, so if everyone could customize it to their own liking, that would probably be the best in my opinion.

P4

#### Gamification

One participant also suggested gamification elements, such as earning points or some kind of praise, or awards in case of good compliance and goal achievement.

I downloaded this app called 'I'm Sober' - it shows you how long you've been sober, and after a few days this little rocket appears. You get kind of excited. And then you see, like, 'Okay, you've hit 100 days or 100 hours,' and this little character pops up and gives you a round of applause. It’s completely silly [laughs]. But somehow it really motivates me to keep using the app [laughs]. Just to see that little creature again.

P10

#### Visualizations

The visualizations presented met with positive reactions. Participants appreciated their potential to contribute to understanding the relationships between their experience and the context in which it takes place.

I would personally find that amazing because effectively that can make you think again of hey, uh that's where I feel unhappy, so that's where I'm wasting my time. But if I want to be happier, then I need to pay more attention. That's super in my mind.

P7

#### Sharing Data With Clinician

All participants found sharing data from the app with their clinician useful. Participants reported that, based on these data, the clinician can better understand their problems and help them more effectively.

I think it's most interesting with a therapist. Someone who has effective insight into and who knows the app very well, as well as the thinking behind it. I also think an app for the individual is important because, as I said, not everyone has [a therapist], but I think that yes, I now have more of the idea that it's actually better with a therapist who really knows the app inside out.

P7

#### Sharing Data With Others

Participants differed in their willingness to share data from the app with their loved ones. Those who state that they have close relationships with others and feel understood by those around them find sharing data useful. Conversely, those who feel misunderstood in relationships are also less willing to share this information.

I personally wouldn't have a problem with that, because I really learned in treatment that honesty is absolutely the number one priority, so also in communication with parents, which I'm trying to improve in my diagnosis, so it would actually be another argument, really indisputable information, it also just sets out how I feel subjectively, but I would take it that it would be some quality output that the parents could see, that I would show them, and they would perhaps then be able to better understand certain situations and conditions if they had that information.

P2

I can imagine that that is useful for some people, but I keep my family and friends very much out of this. My parents didn't know for two years that I was coming to therapy. So... the relationship that I have with the person... but I don't immediately have many people with whom I would share it [my app data]. But I hear from other patients that they do involve more people in their story [therapy process], so that can certainly be useful.

P6

## Discussion

### Principal Findings

This study examined the preferences of patients with problem substance use regarding the content, functionality, and appearance of the DMMH prototype, the use of which is planned as an adjunct to the usual treatment. The findings suggest that patients generally perceive the DMMH prototype positively. They provided several specific suggestions for content, functional and visual changes which are summarized in Textbox 1.

**Recommendations for the design of an Experience Sampling Method–based mobile health tool as an adjunct to usual substance use disorders treatment.**

**Patient-reported design and functionality preferences**

- Up to 10 notifications per day are acceptable, depending on the daily routine and the patient’s condition.
- The questionnaire should be as short as possible.
- The questionnaire should also include open-text response options. These are mainly about circumstances, thoughts, and coping strategies.
- The time window to return to complete the questionnaire should be within 1 hour.
- Incorporate EMI in addition to monitoring.
- Use positive coloring and gamification.
- Visualize data in a simple and clear way.
- Enable data sharing with the clinician.
- Provide a voluntary option to share data with relatives.
- To allow individual customization in all the areas mentioned.

In general, multiple daily notifications were considered acceptable. Participants preferred a number and schedule of notifications that was tailored to their daily routine as well as their current treatment needs. A time window in which to return to the questionnaire was suggested, with 2 participants preferring a time of 1 hour. The acceptability of daily notifications is consistent with the findings of other authors where participants perceived it positively and as a feature that keeps them engaged with the app [33,40]. This could be specific to the group of patients with SUDs, as in studies with patients with other psychiatric diagnoses, 10 notifications per day was considered a rather burdensome amount [41,42]. In a research context, a higher frequency and number of notifications is tolerated if the questionnaire is short [43]. Also, being able to enter the questionnaire when the user unlocks the screen improves compliance [44], which means that it may be advantageous to allow the patient to return to the questionnaire at a later time, but this comes at the cost of not capturing the moment when the notification arrived.

Participants suggested adding to the questions the possibility of recording circumstances, thoughts, or even coping strategies they used. Such a requirement was also a pressing concern in work that surveyed psychiatric patients’ attitudes toward a very similar tool informing the development of the DMMH [42]. Quantifying experience and context alone do not seem to capture all that patients consider important, and noting the specifics of a given situation could increase the potential to understand their patterns of behavior. Self-reflection and resulting insight in people recovering from SUDs are associated with higher treatment engagement, adherence, and abstinence maintenance [45,46]. Recurring suggestions for tracking and promoting healthy lifestyles are consistent with the general popularity of using mobile apps for these purposes [47] and it is an important part of recovery from SUDs [48]. Therefore, it is important to consider this area when designing mHealth tools for individuals with SUDs.

Simple interventions, also called Ecological Momentary Interventions (EMIs), which should be proactively offered to patients based on ESM data, represent a shift from monitoring to intervention and may be useful in relapse prevention [16,49], although the effectiveness of specific kinds of EMIs requires further investigation [50]. If the tool offers such interventions, this contributes to its interactivity, which is a facilitator of user engagement [32,33].

Often, a more colorful design was requested, corresponding to a general preference for a more positive appearance and content for this type of tool [32,33]. Paradoxically, gamification was only suggested by 1 participant. The fact that this option was not mentioned by several participants may be because they were not asked specifically. However, when developing tools of this type, gamification elements should be offered to users as they increase engagement in their use [51]. Of course, whether to use them and, if so, what the gamification features should look like should be discussed with users.

Patients find data visualization useful, especially as a tool to better understand their patterns of behavior and experiences and the contexts in which they take place. Such perceptions of self-monitoring data visualizations are consistent with other studies [52,53]. Well-designed data visualizations can promote patient engagement and compliance, as well as increase the ability of both clinicians and patients to explore, interpret, and discuss data [54,55]. Patients usually best understand number lines and bar graphs, and clear color differentiation is effective in communicating risks, improving understanding and increasing confidence in interpretation [56]. Participants clearly preferred sharing data with their clinicians. They perceived this as an appropriate and valuable addition of information that could help to make treatment more effective. This agrees with the findings of Lyzwinski et al [21], who found that patients in several studies perceived such sharing in a similar way. Willingness to share ESM data with loved ones varied. It depended mainly on the perceived quality of these relationships and a sense of being understood, which is also common when sharing information in other forms in mental health care settings [57].

In terms of content, functionality, and appearance, there were often requests for customization and individualization options for the DMMH. This is consistent with other studies [32,33] and recommendations for increasing user engagement with such tools [31]. Nevertheless, clinicians do not make much use of personalization options, which may be due to time and organizational constraints. Therefore, when developing mHealth tools, it is important to strike a balance between the possibilities of individual personalization and the resulting demands on both the clinician and the patient [42]. Participatory inquiry followed by collaboration of important stakeholders, especially patients, clinicians, researchers, IT and user experience experts, increases the likelihood that the tool will be satisfactory and patients will be more willing to use it [58]. This willingness is enhanced by their early involvement in development [59].

### Implications for Future Research

The results of our study suggest several avenues for further research directions in the development and implementation of ESM-based mHealth tools for people with substance use problems. First, in collaboration with patients and clinicians, look for ways and opportunities to tailor the intensity of monitoring to the patient’s current needs as well as treatment goals. Specifically, consider the patient’s work-family balance and ability to respond to notifications. Also, consider the phase of treatment, where more intensive monitoring with a random schedule may be desirable in the initial phases, after relapse or when changing medication, but may not be necessary and may be burdensome for the patient at the time of remission and stabilization. In such a case, fixed schedule monitoring can be applied. Second, look for technical ways to effectively capture qualitative data to complement quantitative data in ESM protocols and to use it in a meaningful way for the benefit of patients, so that it is not too burdensome for them or for clinicians. Third, explore opportunities for effective linkage between ESM and EMI. This should include proactively offering an intervention based on ESM data, for example, when craving intensifies or intrapsychic tension increases.

The findings highlight the importance of tailoring ESM-based tools to patients’ preferences and treatment contexts. Within the broader IMMERSE project [36], additional qualitative work was conducted to capture the perspectives of other stakeholders, including clinicians, people from the service user support network, and health care administrators involved in implementing such tools for use in general psychiatry setting. However, relatives of service users with problem substance use, clinicians specializing in this group of patients, and health care administrators for people with SUDs were represented only to a limited extent in the project. Future research should therefore ensure their adequate inclusion to better understand how ESM-based interventions can be integrated into clinical settings in a way that meets the needs of all stakeholders in the field of SUD treatment.

### Limitations

Several limitations should be considered when interpreting the results of this study. First, the sample size is relatively small, which may reduce the generalizability of the results. Also, the relatively homogeneous sample in terms of age, with younger and older age groups missing, may have constrained the variability of perspectives. Moreover, the lack of data on race and ethnicity represents an important limitation. Cultural background may shape attitudes toward digital mental health tools, data sharing, and clinician-patient interaction, and these factors could not be examined in this study. Second, there is a risk of selection bias as it can be assumed that participants who are interested in digital technologies will agree to participate in such research and those who are sceptical will also be less willing to participate. Third, there is a risk of bias in terms of social desirability. Although the semistructured format and use of supplementary questions aimed to minimize this bias, participants knew that the interviewers were members of the wider team involved in the development of the DMMH and could therefore provide more positive feedback than they would otherwise have given. In addition, some interviews were conducted by researchers who had previous clinical contact with the participants.

### Conclusions

Participants generally perceived this DMMH positively and expressed a willingness to undergo daily repeated monitoring, especially if one questionnaire takes as little time as possible to complete. This was the sole finding that distinguished individuals with problematic substance use from those with other mental health issues, as all other preferences were similar. Participants considered the possibility of noting down the specifics of situations in the form of open text to be important. Supplementing the monitoring with simple momentary interventions was welcome. Participants considered sharing the collected data with the clinician as beneficial for their collaboration and the success of the treatment. The possibility to adapt the content, the schedule of notifications, and the appearance of the tool to individual needs and preferences was considered important. The study concludes by highlighting the involvement of patients in the development of ESM-based mHealth tools for exploring the alignment of patients’ identified preferences with the realities of clinical practice.

## Appendix

Multimedia Appendix 1 Supplementary Materials 1. IMMERSE Service User Interview Guide.

Multimedia Appendix 2 Supplementary Materials 2. IMMERSE MoMent app prototype.

Multimedia Appendix 3 Supplementary Materials 3. IMMERSE Visualisation Examples.

## Abbreviations

DMMH: Digital Mobile Mental Health
EMI: Ecological Momentary Intervention
ESM: Experience Sampling Method
GDPR: General Data Protection Regulation
ICD-10: International Classification of Diseases, Tenth Revision
IMMERSE: Implementing Mobile Mental health Recording Strategy for Europe
mHealth: mobile health
NASSS: Non-Adoption, Abandonment, Scale-Up, Spread, and Sustainability
SHARE: Scottish Health Research Register
SMHRN: Scottish Mental Health Research Network
SUD: substance use disorder

## Data Availability

The datasets generated or analyzed during this study are available from the corresponding author on reasonable request.
