# Supplementary material for: Preferences for an Experience Sampling Method–Based Tool as an Adjunct to Usual Treatment in Patients With Problem Substance Use: Qualitative Study
Source: JMIR Form Res. 2026 Jan 15;10:e79510. doi: 10.2196/79510 (PMC12856403; doi:10.2196/79510)
Supplement: Multimedia Appendix 1 [file formative_v10i1e79510_app1.pdf]

IMMERSE: Interview guide for service users

V.2, 25.08.2021

Instructions for the interviewer

This interview guide was developed to assist interviewers in conducting a semi-structured interview with participants from the IMMERSE study, phase I.

Read and familiarise yourself with the interview guide before conducting an interview!

All questions should be asked to participants, but if a prompt question has already been addressed by a participant while answering the broad question, the prompt question should not be asked again (skip it). If a participant feels uncomfortable about answering any of the questions, the respective question should be skipped.

Encourage participants to speak as much and as freely as possible, but make sure that the interview stays on track. If a participant tends to talk at length, gently and politely suggest moving to the next question. Try not to interrupt participants or pre-empt their answers, and make sure you understand what they mean by their answers. If you are not sure, ask them to explain. If participants raise important/interesting issues that are not covered by this interview guide, allow for a few minutes to explore the issue further. Use your judgement on whether participants' responses are relevant to the study aims, and gently steer participants back to the topic guide if necessary.

The interviewer can choose to make minor changes to the phrasing of the questions to make it more natural, but the content and meaning of the questions should not be changed.

The interview should take approximately 45 - 60 minutes. Keep track of time, to make sure that you make it through this interview guide within the time. If you notice that you might need a few more minutes, check with the participant whether they are happy to talk to you for a little bit longer.

Interview guide

Introduction and practical information

My name is ______ , I am working as a researcher with the IMMERSE study team, and I will be conducting this interview today.

First of all, thank you for taking the time to talk to me. You sharing your experience is very valuable for us to understand the daily life context in which DMMH will be used and how we can improve the DMMH app. Please remember there are no ‘right’ or ‘wrong’ answers, we would simply like to hear more about your experiences and views in relation to using mental health apps.

In this interview, we will discuss different themes. We will focus on your previous experience with and potential future use of mental health apps, your opinion on a prototype of the DMMH app, how it would be for you to use it, and the impact it may have on you(r therapy) and your daily life.

This interview will be audio recorded. The recordings will be saved using an anonymized study ID and transcribed into text by a team member, which can then be used for analysis.

The interview will take approximately 45 - 60 minutes. If you would like a break at any point, please let me know.

Are you happy for me to record this interview? This is so we remember all the details of our discussion. (If yes, **put on voice recorder**.)

Do you have any questions at this point? If not, I will start the interview.

You have been invited to take part in this interview as you responded to our invitation (ad/call for participants).

We are interested in speaking to a wide range of people, so we ask participants a few questions about their background.

Age:

Gender:

Ethnicity:

Employment status:

Education:

Area of residence/city/health board:

I would like to learn a bit more about your **previous experience with mental health apps**.

1. If you feel comfortable, could you please tell me about your current experience with accessing mental health treatment? (How long? Diagnosis?)

2. Have you ever used any app for mental health treatment? (Or used any other self-monitoring apps? Why (not)? If yes, which ones did you use? Are you still using them? If not, why did you stop?)

I would like to show you a **prototype of the DMMH app** and talk about your views on using it. [Interviewer to explain about how the app works/its key aims].

I would like to know a bit more about your views of the app and your opinion on the questions that it asks you to complete at each beep.

3. What do you think about the app? What do you like/dislike?

4. What would it be like for you paying attention to your feelings multiple times a day?

5. What do you think of the questions in the app?

    a. Do you find them relevant?

    b. Are they easy to understand and answer to?

6. If you could **make any changes to** the app (e.g. adding questions or answer options), what would you do?

7. Is there anything else that you would like to monitor in relation to your mental health? Anything else you would like to see included in the app? [e.g.physical activity, food, sleep]

I would like to talk about notifications/alerts now. I've got an example (vignette) to illustrate this.

> Thomas works as a nurse, and he is mostly on night shifts. While doing his self-monitoring period using DMMH, the app would send him regular reminders throughout the day. This would disturb his sleep, and he would feel guilty (and a bit annoyed) about not completing the questions at each notification.
>
> The app does have a customisation option, so together with his clinician, Thomas was able to change the time he would get alerted to be more suitable for his work pattern.

8. How do you feel about using alerts and reminders on your phone?

9. How would you feel about being alerted 7-10 times per day?

Next, we are going to talk about how using the DMMH app might fit into people's daily routine.

10. How much **time** would you find acceptable for completing the questions? [Would finding time be a challenge for you?]

11. How do you think using the app would **fit into your daily routine**? What would be **barriers** to using the app/completing the questions? [prompts: making any changes to your routine to respond to notifications, inconvenient situations]

12. Do you sometimes experience any **technical problems** while using your phone/tablet (e.g. connectivity issues, screen freeze, glitches)?

We're about halfway through the interview. I am now going to ask you some questions on the **possibilities DMMH offers in relation to data feedback/discussion with your clinician** and the impact that using the DMMH app may have on you, your therapy and/or your clinician.

First, here is an example of how the DMMH app could work in practice.

Mark took out early retirement due to his health. He used the DMMH app to get an insight into whether his current treatment is working. It became very clear to him that a major factor for his mental health is stress. He found out more about triggers that make him feel stressed. He said that “using the app showed me that I am not good at coping with stress.” He was able to address this with his clinician and they discussed various ways that can help him cope when he feels very stressed. These included behavioural interventions such as doing more physical activity and spending time outdoors.

What do you think about discussing your results from the app with your clinician?

14. How do you think that DMMH might affect your ongoing therapy, and your role in therapy? Would it help you feel more involved in decisions about your clinical care? If yes, how?

15. How would it improve the quality of support you receive from your clinician? (If so)

We are now going to discuss people’s motivation to use self-monitoring apps such as DMMH, starting with an example (vignette).

Nathan is a 27-year-old business analyst. He used to write down notes about his symptoms and feelings but completing the app has given him a better understanding. He said: “I usually write it down every day, but that is more superficial. Thanks to the app, I can cover this more deeply and get help, that is what I like about it. It is also easy to send to my clinician.” He acknowledges that it takes more time to complete the app at each alert, but it has helped him getting a more personalised treatment in his therapy.

What would motivate you to use self-monitoring apps such as DMMH? (e.g. learning something new about yourself/your mental health?)

I would like to know a bit more about the **impact that using the DMMH app may have on your daily life.**

17. Would you change (or are you thinking about changing) your usual routine/behaviour based on what you could learn about yourself by using the app?

18. How might using self-monitoring apps/DMMH, help you cope differently/better with your symptoms? (If so)

19. After seeing a prototype of the DMMH app, will it solve any challenges you are currently experiencing? If yes, can you give an example? If no, why not?

We have come to the last part of the interview. I would like to talk very briefly about **possible future use of digital health apps** and any suggestions for improvement.

20. Would you like to use DMMH (or similar self-monitoring apps) in the future? Why (not)? If yes, would you like to use the app independently, or do you prefer to have it integrated in your therapy?

21. How would you feel about sharing information you've entered into the app with others such as friends/family?

22. Is there anything you would change about the DMMH app? What and why?

23. Is there anything else you would like to add? Do you have any questions?

Thank you again for your time. We hope you enjoyed participating in this study. If you have any further questions regarding the study, you can always contact us on our email address or phone number.
