# Supplementary material for: Preferences for an Experience Sampling Method–Based Tool as an Adjunct to Usual Treatment in Patients With Problem Substance Use: Qualitative Study
Source: JMIR Form Res. 2026 Jan 15;10:e79510. doi: 10.2196/79510 (PMC12856403; doi:10.2196/79510)

IMMERSE Visualisation Examples

Name	**Personal Therapy Goal**
Description	Perceived progress in service-users’ personal therapy goals is assessed once per day, as a part of the evening questionnaire. For each goal, two items assess the perceived progress made that day and service-users satisfaction with the progress. This data is visualized in a line graph, in which a trend line can be added.
Visualization	**Personal Goal Progress** 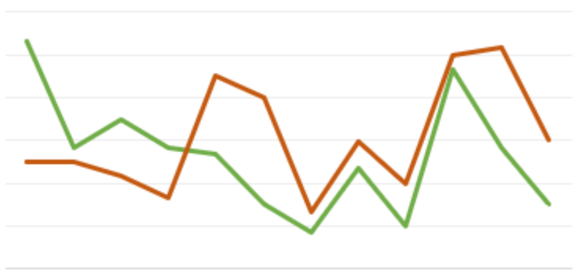

<table>
<tr><td>Name</td><td>Activity Context</td></tr>
<tr><td>Description</td><td>An overview of the service-user’s daily activities can be obtained from the activity pie chart, summarizing and displaying the distribution of the different reported activities. Users can choose the time frame for which they want to have data displayed (e.g. last 7 days) . Summarized ranking of activities with the highest appraisal is also displayed.</td></tr>
<tr><td>Visualization</td><td>
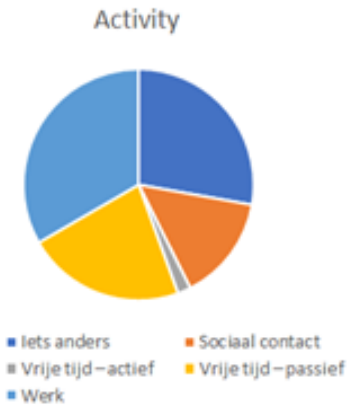

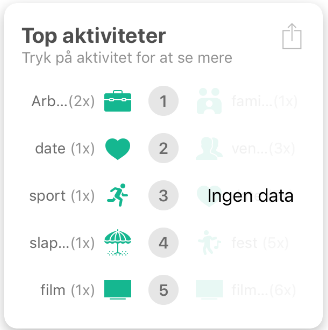

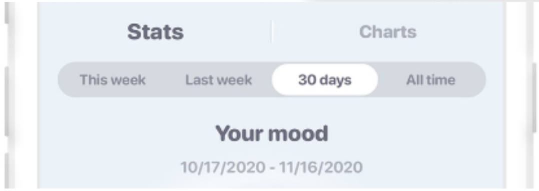

(Example time frame selection from “moodnotes’’)
</td></tr>
</table>

Activities vs. mood

25.06.2021-29.06.2021

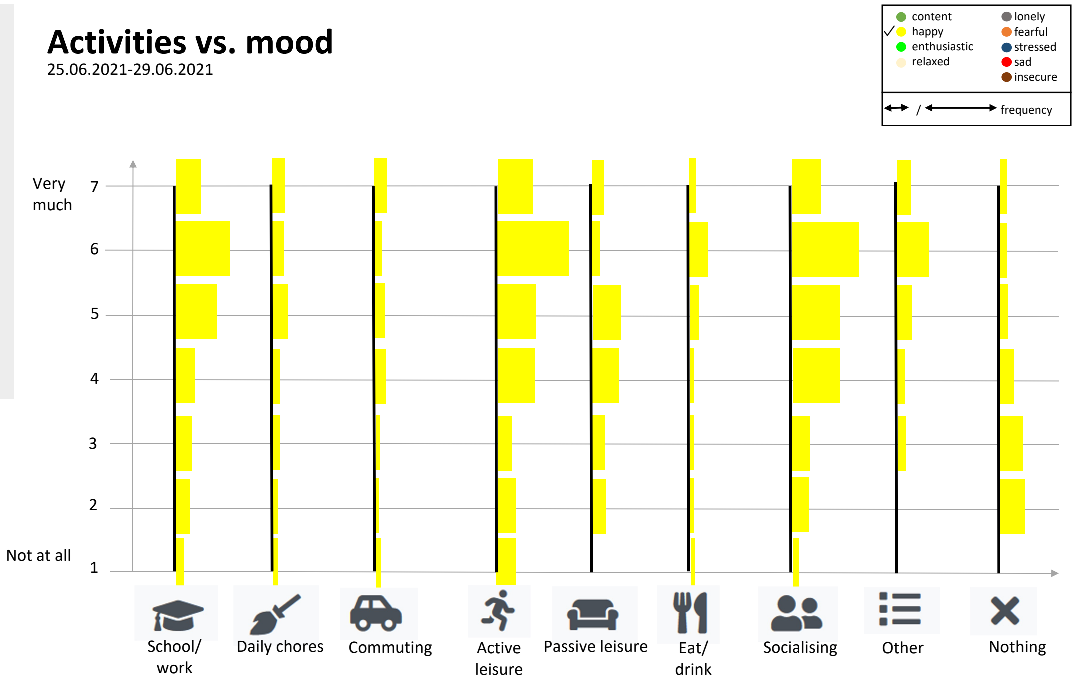

Supplement: Multimedia Appendix 3 [file formative_v10i1e79510_app3.pdf]
